# An unusual cause of progressive dyspnoea, pulmonary nodules and pleural effusions

**DOI:** 10.1002/rcr2.1261

**Published:** 2023-11-30

**Authors:** Sudharsan Venkatesan, Bharti Ramnani, Sonja Klebe, Lin Mo

**Affiliations:** ^1^ Division of Medicine Royal Darwin Hospital Darwin Northern Territory Australia; ^2^ College of Medicine and Public Health Flinders University Darwin Northern Territory Australia; ^3^ Department of Anatomical Pathology Royal Darwin Hospital Darwin Northern Territory Australia; ^4^ Department of Anatomical Pathology Flinders Medical Centre Adelaide South Australia Australia

**Keywords:** ANCA‐negative EGPA, nodules, pleural effusions

## Abstract

Eosinophilic granulomatosis with polyangiitis (EGPA) is part of the ANCA‐associated vasculitides, although ANCA‐negative presentations are not infrequent. Asthma, allergic rhinitis and peripheral eosinophilia are classic associations although pleural effusions are an uncommon manifestation of EGPA. We describe a 35‐year old Caucasian adult with asthma who presented with progressive dyspnoea, cough, peripheral eosinophilia and imaging that showed pulmonary nodules with pleural effusions. Biopsy with histopathological review confirmed the diagnosis of EGPA with steroid therapy resulting in significant resolution of symptoms and imaging findings.

## Graphical abstract

The multiple radiological changes associated with Eosinophilic Granulomatosis with Polyangiitis.

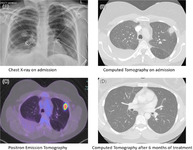

## INTRODUCTION

Eosinophilic Granulomatosis with Polyangiitis (EGPA) is a multi‐organ disease and can present with a myriad of clinical manifestations, with or without anti‐neutrophil cytoplasmic antibody (ANCA) positivity. A high index of suspicion is required to diagnose this condition using clinical, histopathological and radiologic features given the overlap of clinical conditions with similar phenotypes. An accurate diagnosis is required for appropriate immunosuppressive therapy. In this report, we describe the presentation and management of a patient with ANCA‐negative EGPA.

## CASE REPORT

A 35‐year old Caucasian woman was admitted to a tertiary healthcare centre in the Northern Territory of Australia after multiple emergency department presentations with left sided pleuritic chest pain, dry cough, dyspnoea and subjective fevers. Her medical history was significant for asthma on Salmeterol/Fluticasone, allergic rhinitis and previous provoked pulmonary embolism in the post‐partum period. She did not take any other medications and her exposure history was unremarkable apart from working in the cosmetics industry. She was a nurse who resided in a rural property outside of Darwin (Northern Territory, Australia) with her husband and three children. She did not have any significant bird or animal contact. She wore closed shoes when outdoors and had no contact with potting mix or soil. She had no history of cigarette or recreational drug use.

**FIGURE 1:**
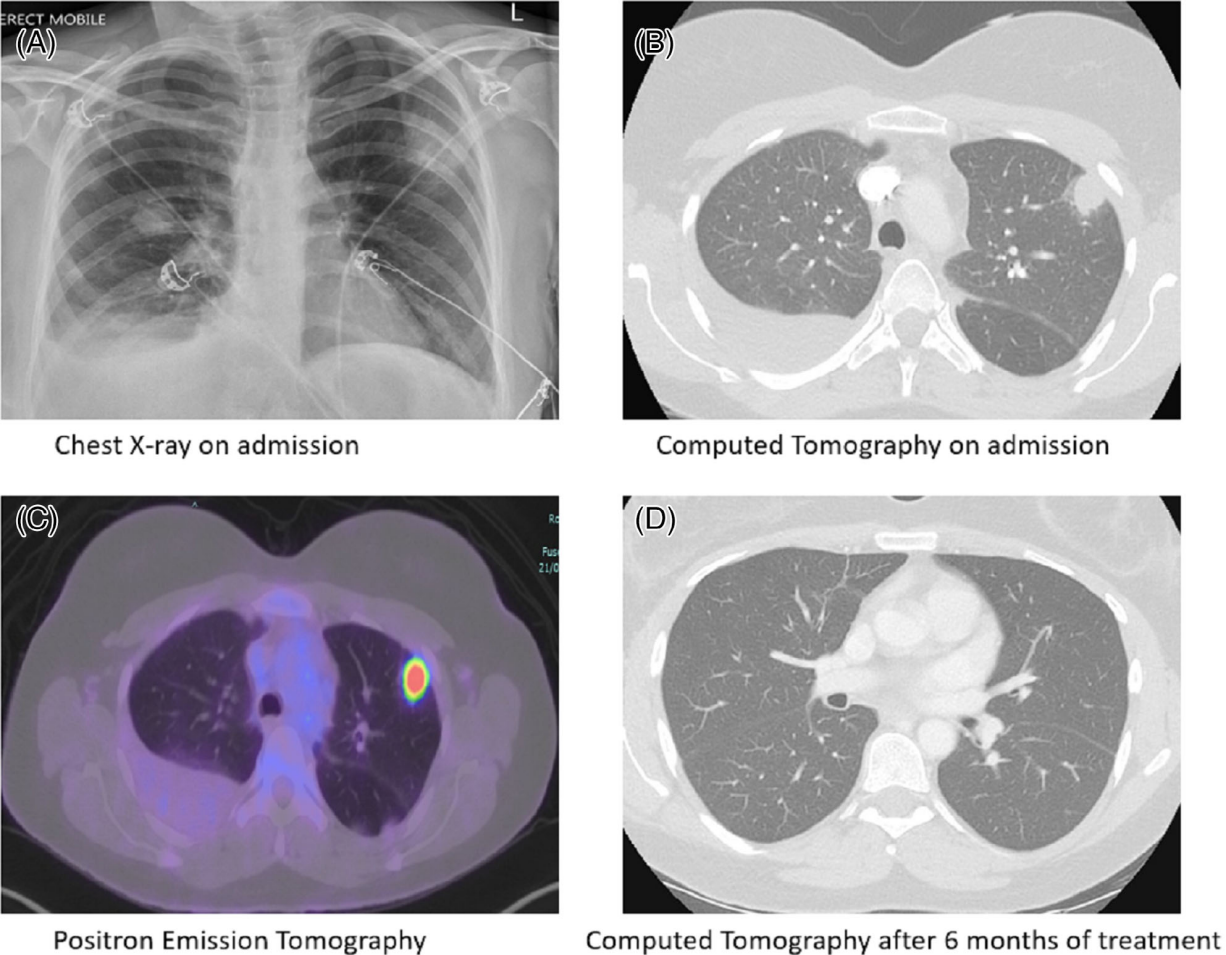
Chest imaging with various modalities at distinct phases of patient presentation.

A Computed Tomography Pulmonary Angiogram (CTPA) on admission demonstrated multifocal bilateral rounded consolidation with small bilateral pleural effusions (Figure 1). Laboratory investigations revealed peripheral eosinophilia (1.5 × 10^9^/L) and an elevated erythrocyte sedimentation rate. Urinalysis was normal and no growth was detected on multiple blood cultures. Serology revealed elevated *Legionella longbeachae* Indirect Fluorescent Antibody titre and raised *Mycoplasma pneumoniae* IgM with negative IgG (Tables 1 and 2).

**TABLE 1:** Summary of investigations at presentation and other relevant time‐points.

Investigation (unit)	Initial presentation	Prior to immunosuppression	2 weeks after immunosuppression	12 months after completion of immunosuppression	Reference range
Haemoglobin (g/L)	114	104	117	124	115–165
White cell count (×10^9^/L)	11.3	8.9	11/2	7.9	4.0–11.0
Eosinophil count (×10^9^/L)	1	0.7	0	0.7	0.0–0.4
Serum creatinine (umol/L)	49	56	72	67	45–90
C‐reactive protein (mg/L)	70	43	1	2	<5
Erythrocyte sedimentation rate (mm/h)	73	79	17	32	0–25

**TABLE 2:** Summary of infectious and non‐infectious investigations.

Serological investigations (method)	Initial	Convalescent (if available)
Infectious aetiology		
Aspergillus Galactomannan	Not detected	
Aspergillus specific IgG	Negative	
Bartonella henselae (IFA)	Not detected	
Burkholderia pseuodomallei (IHAT)	Negative	Negative
Brucella IgM and IgG (EIA)	IgM detected	
Chlamydophila pneumoniae IgG (EIA)	Negative	
Cryptococcus Antigen (Lateral Flow Assay)	Negative	Negative
Cytomegalovirus IgM and IgG	IgG detected	IgG detected
Ebstein Barr Virus IgM and IgG (EBNA)	IgG detected	
Filaria IgG	Not detected	
Hepatitis B Virus	Immune with no evidence of past infection	
Hepatitis C Antibody	Not detected	
Human Immunodeficiency Virus Antigen/Antibody	Not detected	
Human T‐Lymphotrophic Virus I/II Antibody (CMIA)	Not detected	
Legionella pneumophila (IFA)	Negative	Negative
Legionella longbeachae (IFA)	**Detected (2048)**	**Detected (2048)**
Mycoplasma pneumoniae IgM	**Positive**	**Positive**
Mycoplasma pneumoniae IgG	Negative	Negative
Q Fever Phase 2 IgM and IgG (IFA)	Negative	
Q Fever Phase 1 IgG (IFA)	Negative	
Quantiferon‐TB Gold Plus	Negative	
Strongloides IgG (EIA)	Not detected	Not detected
Syphilis Antibody Screen	Not detected	
Toxocara IgG ratio	Not detected	
Toxoplasma gondii IgM and IgG	Not detected	
**Auto‐immune, vasculitic and rheumatological aetiology (units)**	**Result**	**Reference Range**
Angiotensin converting enzyme	29	20–70
Antiphospholipid syndrome studies	Negative	
Anti‐cyclic citrullinated peptide (U/mL)	1	<5
Anti‐double stranded DNA antibody (IU/mL)	<7	0–7
Anti‐nuclear antibody	Negative	
Anti‐neutrophil cytoplasmic antibodies (c‐ANCA and p‐ANCA)	Negative	
C3 Complement (g/L)	1.17	0.86–1.84
C4 Complement (g/L)	**0.06**	0.20–0.59
Extractable nuclear antigen antibodies	Negative	
Immunoglobulins IgM, IgA, and IgG (g/L)	Normal	
Immunoglobulin E (kIU/L)	**60**	<26
Rheumatoid factor (kIU/L)	**31.1**	0.0–12.0

*Note*: Bold indicates abnormal result.

She was initially treated with a 7 day course of oral amoxicillin and doxycycline as well as dexamethasone. After initial improvement her symptoms relapsed and she was readmitted with a large right sided pleural effusion requiring pleurocentesis. A large volume pleurocentesis was an exudate by Light's criteria (pleural fluid Lactate Dehydrogenase 321 U/L) however, the specimen was clotted, precluding an accurate cell count. There was no growth on standard bacterial and mycobacterial cultures. Adenosine deaminase was not elevated. Pleural fluid cytology revealed an inflammatory exudate and she proceeded to a Fluorodeoxyglucose (FDG) Positron Emission Tomography (PET) scan (Figure 1), diagnostic bronchoscopy and CT guided transthoracic biopsy of her peripheral mass like consolidation. FDG PET revealed increased uptake within the pulmonary lesions but no sites of distant disease. No organisms were isolated (via standard and selective cultures) or detected (via nucleic acid amplification testing) from the bronchoscopy. Histology from the biopsy revealed scattered non‐necrotizing granulomas, abundant histiocytes and focal eosinophilia and a differential of hypersensitivity pneumonitis (HP) was raised. A second histology opinion was sought as the clinical and radiological picture was inconsistent with HP.

**FIGURE 2:**
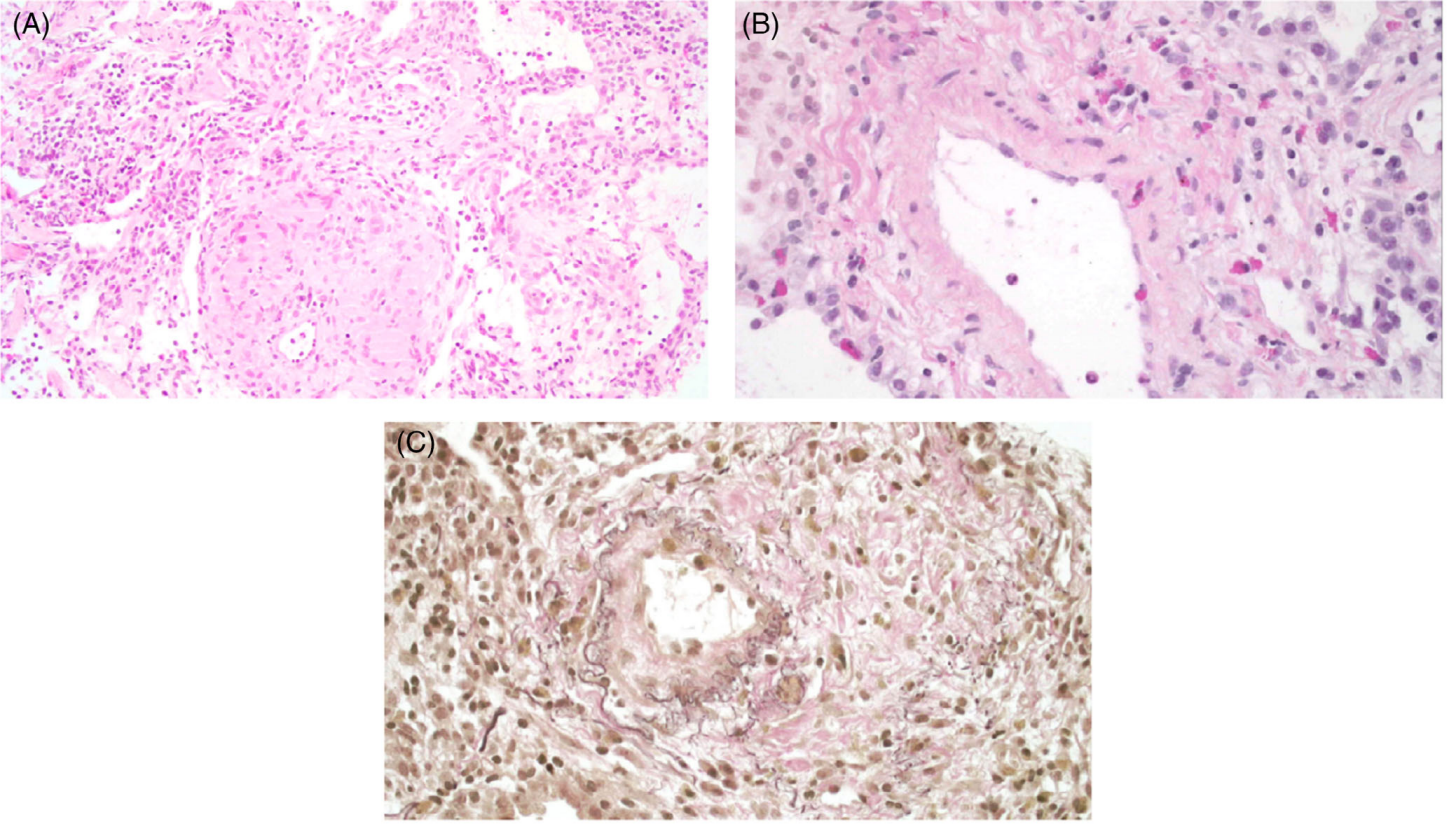
H&E‐stained section of the bronchial biopsy shows a peribronchial granuloma (2A), H&E‐stained section of the bronchial biopsy shows eosinophil‐rich vasculitis with eosinophils infiltrating (2B), VVG stain demonstrates disruption of elastic lamina (2C).

She represented with recurrent pleural effusion and dyspnoea and repeat CT Chest demonstrated progression of pulmonary lesions. The second histology opinion suggested a diagnosis of EGPA with disruption of the elastic lamina seen on Verhoeff‐Van Gieson (VVG) stain (Figure 2). Anti‐neutrophil cytoplasmic antibodies (ANCA) were negative. She was commenced on prednisolone 50 mg daily with a calculated Five‐Factor Score of 0, as well as a course of azithromycin given Legionella serology results. A repeat chest x‐ray 2 weeks later demonstrated resolution of pleural effusions and significant improvement in pulmonary opacities with almost complete resolution of her symptoms. Azathioprine was commenced for steroid sparing effect and immunosuppression was continued for 1 year. No relapses were noted 1 year following cessation of therapy.

## DISCUSSION

EGPA is a multisystem disorder involving small and/or medium vessels and is a part of the ANCA‐associated vasculitides (AAV).^1^ As in our case, EGPA occurs in patients with asthma, allergic rhinitis and peripheral eosinophilia and can present with peripheral neuropathy, pulmonary opacities, worsening asthma and rash among other symptoms.

In one retrospective review of 348 patients diagnosed with EGPA, 9% of patients in this cohort study had pleural effusions but this was more frequent in ANCA‐negative patients compared to ANCA‐positive patients (11.7% vs. 4.6%, respectively).^2^ In another study, 61% of the ANCA negative EGPA cohort had migratory lung infiltrates however, only 3% had pleural effusions suggesting that pleural effusions are a rare presentation.^3^

The hallmark of histological findings in EGPA are necrotising small vessel vasculitis accompanied by eosinophilic infiltrates and perivascular and extravascular granulomas.^4^ In contrast to our case, biopsy‐proven vasculitis is found more frequently in ANCA‐positive patients than ANCA‐negative patients, although eosinophilic infiltrates and granulomas are found in similar rates in both groups.^3^

Important differentials include acute and chronic eosinophilic pneumonia, parasitic infections, Hyper‐Eosinophilic Syndrome (HES), Allergic Bronchopulmonary Aspergillosis (ABPA) and drug induced eosinophilic lung disease.^5^ In our case *L. longbeachae* infection was considered as an unlikely cause as the clinical picture did not fit, however the patient received treatment with azithromycin along with steroids. The elevated *Mycoplasma* IgM was thought to be of no clinical consequence and likely due to cross reactivity. Concurrent parasitic infection was considered but clinically excluded as there were no abnormal results with serology‐based testing for parasites (Table 2), there were no calcifications or cystic changes on imaging, repeat histopathological examination of the biopsied pulmonary nodule did not identify features of parasitic infection (such as focal ulcers or characteristic ova) and there was significant resolution of clinical and radiographic changes without parasite‐specific treatment.

In conclusion, EGPA presents with a broad range of symptoms and especially in ANCA negative cases, the presentation may be atypical. For diffuse pulmonary infiltrates with or without pleural effusions, if there is a history of asthma or allergic rhinitis with associated peripheral eosinophilia, the diagnosis of EGPA should always be considered.

## AUTHOR CONTRIBUTIONS

**Sudharsan Venkatesan**: Preparation of manuscript; subsequent drafts. **Bharti Ramnani**: preparation of histology figures; manuscript review. **Sonja Klebe**: preparation of histology figures; manuscript review. **Lin Mo**: manuscript revision and review.

## CONFLICT OF INTEREST STATEMENT

None declared.

## ETHICS STATEMENT

The authors declare that appropriate written informed consent was obtained for the publication of this manuscript and accompanying images.

## Data Availability

Data sharing not applicable to this article as no datasets were generated or analysed during the current study.
